# Molecular Hydrogen Ameliorates Anti-Desmoglein 1 Antibody-Induced Pemphigus-Associated Interstitial Lung Disease by Inhibiting Oxidative Stress

**DOI:** 10.3390/ijms26094203

**Published:** 2025-04-28

**Authors:** Chang Tang, Lanting Wang, Zihua Chen, Xiangguang Shi, Yahui Chen, Jin Yang, Haiqing Gao, Chenggong Guan, Shan He, Luyao Zhang, Shenyuan Zheng, Fanping Yang, Sheng-An Chen, Li Ma, Zhen Zhang, Ying Zhao, Qingmei Liu, Jiucun Wang, Xiaoqun Luo

**Affiliations:** 1 Department of Allergy & Immunology, Huashan Hospital, Fudan University, Shanghai 200437, China; 15301050176@fudan.edu.cn (C.T.); lteer.w@hotmail.com (L.W.); tzuhuac@163.com (Z.C.); aurorayangj@163.com (J.Y.); ghq0810@163.com (H.G.); gcg1008u@gmail.com (C.G.); shawhe16@163.com (S.H.); zhangluyaozly@sina.com (L.Z.); zsysmiling@163.com (S.Z.); fanpingyang@126.com (F.Y.); hikaru_csa@hotmail.com (S.-A.C.); 13817520375@139.com (L.M.); ximalaya3@yeah.net (Z.Z.); zhaoying421@hotmail.com (Y.Z.); 2 Department of Dermatology, Huashan Hospital, Fudan University, Shanghai 200437, China; guangxiangshi@163.com (X.S.); liuing.mei@163.com (Q.L.); jcwang@fudan.edu.cn (J.W.); 3 Research Center of Allergy and Diseases, Fudan University, Shanghai 200437, China; 4 State Key Laboratory of Genetic Engineering, Collaborative Innovation Center for Genetics and Development, School of Life Sciences, and Human Phenome Institute, Fudan University, Shanghai 200437, China; 21112030003@m.fudan.edu.cn

**Keywords:** molecular hydrogen, anti-desmoglein 1 antibody, pemphigus, interstitial lung disease, oxidative stress

## Abstract

Pemphigus-associated interstitial lung disease (P-ILD) is a severe complication observed in pemphigus patients that is characterized by pulmonary interstitial inflammation and fibrosis. This study investigated the role of anti-desmoglein (Dsg) 1/3 antibodies in P-ILD pathogenesis and evaluated the therapeutic potential of molecular hydrogen (H_2_). Using a BALB/cJGpt mouse model, we demonstrated that anti-Dsg 1 antibodies, but not anti-Dsg 3 antibodies, induced interstitial inflammation and fibrosis. Immunofluorescence staining confirmed IgG deposition in the alveolar epithelium, suggesting immune complex formation and epithelial damage. Gene expression analysis revealed elevated pro-inflammatory cytokines (*IL-1β*, *IL-13*) and upregulated pro-fibrotic markers (*α-SMA*, *S100A4*, *TGF-β*, and collagen genes) in P-ILD progression. Elevated oxidative stress and impaired ROS metabolism further implied the role of oxidative damage in disease pathogenesis. To assess H_2_’s therapeutic potential, hydrogen-rich water was administered to P-ILD mice. H_2_ treatment significantly reduced oxidative stress, attenuated interstitial inflammation, and prevented pulmonary fibrosis. These protective effects were attributed to H_2_’s antioxidant properties, which restored the pro-oxidant–antioxidant balance. Our findings underscore the critical role of anti-Dsg 1 antibodies and oxidative stress in P-ILD and highlight H_2_ as a promising therapeutic agent for mitigating anti-Dsg 1 antibody-induced lung injury.

## 1. Introduction

It was previously believed that pemphigus was an organ-specific autoimmune skin disease that only involves the skin and mucosa [1,2]. Previous studies of pemphigus complications mainly focused on the side effects of steroids and immunosuppressants [3]. However, through the statistical analysis of 573 patients with pemphigus, Chen et al. found that interstitial lung disease (ILD) was one of the most prevalent comorbidities in post-diagnosis pemphigus cases and the leading cause of death for the cohort [4]. Furthermore, Tang et al. established a mouse model of pemphigus by the subcutaneous injection of serum immunoglobulin G (IgG) from patients with pemphigus in mice and found that there were interstitial lung lesions in the lungs of pemphigus mice [5]. This suggested that there might be a correlation between pemphigus and ILD and that pemphigus patients with ILD could be considered as suffering from pemphigus-associated interstitial lung disease (P-ILD).

More importantly, Tang et al. found that there was serum-derived IgG deposition in the lungs of pemphigus mice, suggesting that this interstitial change might be related to the direct effect of the subcutaneous injection of human IgG on the lung tissues of mice [5]. Several case studies have reported that IgG deposition was found in the bronchoalveolar epithelium of patients with pemphigus lung involvement and that the desmoglein (Dsg) antigen was ectopically expressed in lung tissue [6,7,8,9]. Therefore, we speculate that the high titer of anti-Dsg 1/3 antibodies in the serum of patients with pemphigus may act on the lung epithelial tissue to cause lung injury, resulting in ILD in patients with pemphigus. Exploring the pathogenesis of P-ILD could be of great significance for the early detection of and intervention in this condition.

Oxidative stress plays a key role in the development of ILD [10]. The production of reactive oxygen species (ROS) is responsible for oxidative stress, which induces inflammation in the body, which, in turn, activates the proliferation of lung fibroblasts to produce collagen deposition, and, ultimately, forms pulmonary fibrosis [10]. Because pulmonary fibrosis is irreversible, effective intervention in the early inflammatory stage is considered to be an important means of treating ILD [11]. N-acetylcysteine (NAC) is a precursor of reduced glutathione, which can scavenge oxygen free radicals in the body [12]. As a commonly used antioxidant, it has been clinically used to treat early-stage ILD patients [13,14,15]. Nonetheless, the bioavailability of orally administered NAC in pulmonary tissue is limited, and its non-specific scavenging of oxygen free radicals may inadvertently eliminate ROS with significant signaling effects [16,17]. This could lead to a disruption in the body’s redox balance, thereby constraining its therapeutic efficacy. Two recent randomized controlled trials failed to observe the significant survival benefit of NAC monotherapy in ILD, suggesting that we need more effective and safer antioxidant drugs to treat early ILD [18,19].

Molecular hydrogen (H_2_), formed by two hydrogen atoms tightly connected by covalent bonds, is the lightest and smallest gas molecule [20]. Benefiting from its low density, strong permeability, and fast diffusion speed, H_2_ can directly reach the vicinity of the mitochondria responsible for producing ROS in cells [20]. The most cytotoxic ROS are selectively reduced, including hydroxyl radicals (·OH) and peroxynitrite (ONOO-), without interfering with metabolic redox reactions [21]. Consequently, it has become accepted practice to treat many diseases with an efficient and harmless antioxidant [22,23]. Therefore, we believe that H_2_ may have a good therapeutic effect on P-ILD. The early detection and timely treatment of P-ILD are of great significance for improving the prognosis of patients with pemphigus.

## 2. Results

### 2.1. Anti-Dsg 1 Antibody-Induced Interstitial Lung Disease in the Pemphigus Mouse Model

**Figure 1:**
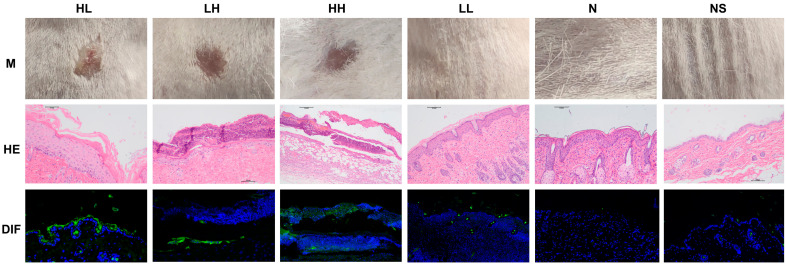
The pemphigus mouse model was established by the subcutaneous injection of high-titer anti-Dsg 1/3 antibody serum IgG. M shows the effects on mouse skin; HE shows the hematoxylin-eosin staining of mouse skin (microscope magnification of 100×); DIF shows the direct immunofluorescence staining of mouse skin (fluorescence microscope magnification of 40×); HL—mice were subcutaneously injected with high anti-Dsg 1 antibody/low anti-Dsg 3 antibody titer of pemphigus patient serum, IgG group; LH—mice were subcutaneously injected with low anti-Dsg 1 antibody/high anti-Dsg 3 antibody titer of pemphigus patient serum, IgG group; HH—mice were subcutaneously injected with high anti-Dsg 1 antibody/high anti-Dsg 3 antibody titer of pemphigus patient serum, IgG group; LL—mice were subcutaneously injected with low anti-Dsg 1 antibody/low anti-Dsg 3 antibody titer of pemphigus patient serum, IgG group; N—mice were subcutaneously injected with normal healthy human serum IgG group; NS—mice received a subcutaneous injection of the normal saline group. IgG deposition (green); DAPI, nucleus (blue).

The pemphigus mouse model was successfully established in the HL, LH, and HH groups by administering subcutaneous injections of serum IgG with varying anti-Dsg 1/3 antibody titers to BALB/cJGpt mice (Figure 1).

**Figure 2:**
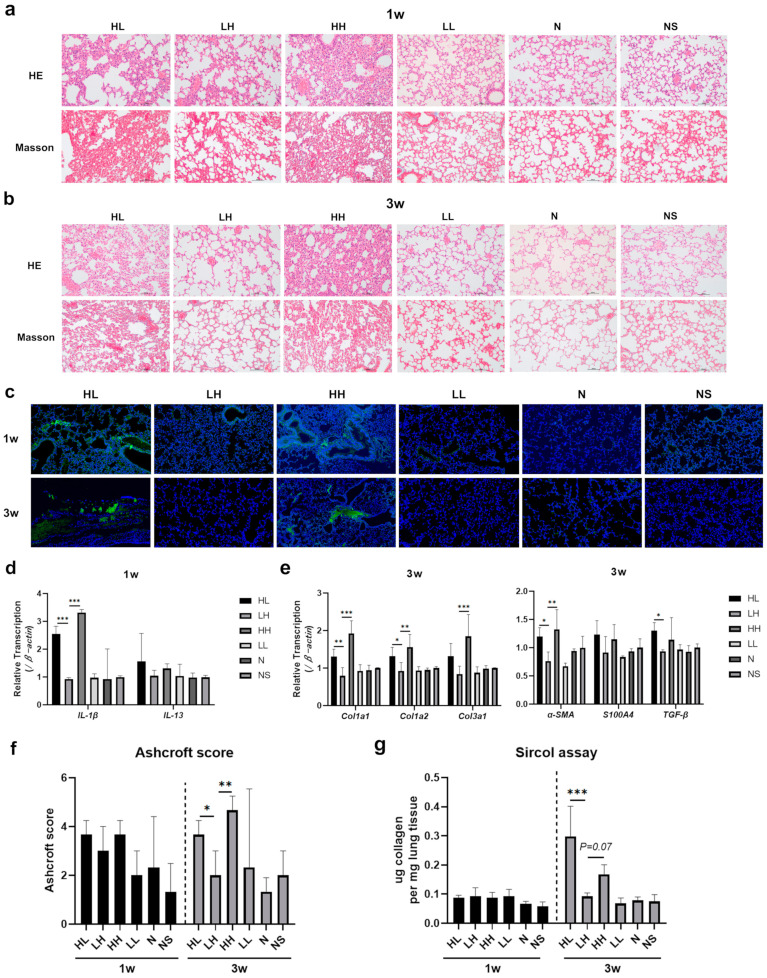
Anti-Dsg 1 antibody-induced interstitial lung disease in mice. (**a**) HE staining and Masson staining of lung tissue in mice after 1 week (microscope magnification of 100×); (**b**) HE staining and Masson staining of lung tissue in mice after 3 weeks (microscope magnification of 100×); (**c**) direct immunofluorescence staining of mouse lung tissue (fluorescence microscope magnification of 20×); (**d**) RT-qPCR was used to detect the changes in *IL-1β* and *IL-13* gene expression in the lung tissue of mice after 1 week; (**e**) RT-qPCR was used to detect the changes of *α-SMA*, *S100A4*, *TGF-β*, *Col1a1*, *Col1a2* and *Col3a1* gene expression in lung tissue of mice at 3 weeks; (**f**) Ashcroft score of lung tissue in mice; (**g**) Collagen content in lung tissue of mice in each group was detected by Sircol assay. IgG deposition (green); DAPI, nucleus (blue). Data were analyzed using a one-way ANOVA with Bonferroni’s post hoc test for multiple comparisons. The histogram represents mean ± standard deviation; * *p* < 0.05, ** *p* < 0.01, and *** *p* < 0.001.

Lung tissues from each group were collected for histopathological examination after 1 week. HE staining revealed alveolar septal thickening, local lung tissue destruction, and significant inflammatory cell infiltration in the lung tissues of mice in the HL and HH groups. In comparison, the inflammatory response in the LH group was less severe, with localized inflammatory cell infiltration observed. No discernible pathological alterations were observed in the lung tissues of the LL, N, and NS groups. Masson staining revealed slight fibroblast proliferation in the alveolar space of mice in the HL and HH groups, with no comparable findings in the LH, LL, N, and NS groups (Figure 2a). After 3 weeks, fibroblast proliferation and collagen fiber deposition were more pronounced in the lung tissue of mice in the HL and HH groups compared to the LH, LL, N, and NS groups (Figure 2b). Furthermore, lung tissue samples were obtained from pemphigus mice post-modeling for direct immunofluorescence analysis. The results revealed the presence of IgG deposition in the alveolar epithelium of HL and HH groups, with a notable increase observed after 3 weeks compared to deposition after 1 week. Conversely, no IgG deposition was detected in the lung tissue of LH, LL, N, and NS groups at either time point (Figure 2c).

Consistently, the RT-qPCR results showed that the expression of pro-inflammatory factors *IL-1β* and *IL-13* increased in the HL and HH groups at 1 week, while the expression of the other four groups did not change (Figure 2d). After 3 weeks, compared with the LH, LL, N, and NS groups, the expression of pro-fibrotic genes (*α-SMA*, *S100A4*, *TGF-β*) and collagen genes (*Col1a1*, *Col1a2*, *Col3a1*) in the lung tissue of mice in the HL and HH groups was significantly increased (Figure 2e). The Ashcroft score was employed for the semi-quantitative assessment of lung fibrosis in mice. No notable fibrosis was observed in the lungs of mice in any group after 1 week. There were no significant disparities in the Ashcroft score and Sircol collagen content among the groups. In contrast, after 3 weeks, pulmonary fibrosis exhibited a marked increase in severity in the HL and HH groups (Figure 2f,g).

These findings suggested that the subcutaneous administration of the anti-Dsg 1 antibody in mice could trigger an interstitial inflammatory response in the lungs after 1 week, followed by the development of pulmonary fibrosis by the 3-week mark. The presence of inflammation and fibrosis in the lungs of P-ILD mice was linked to the accumulation of immune complexes generated by anti-Dsg 1 antibodies.

### 2.2. Anti-Dsg 1 Antibody Induced Increased Oxidative Stress in the Lungs of Mice

**Figure 3:**
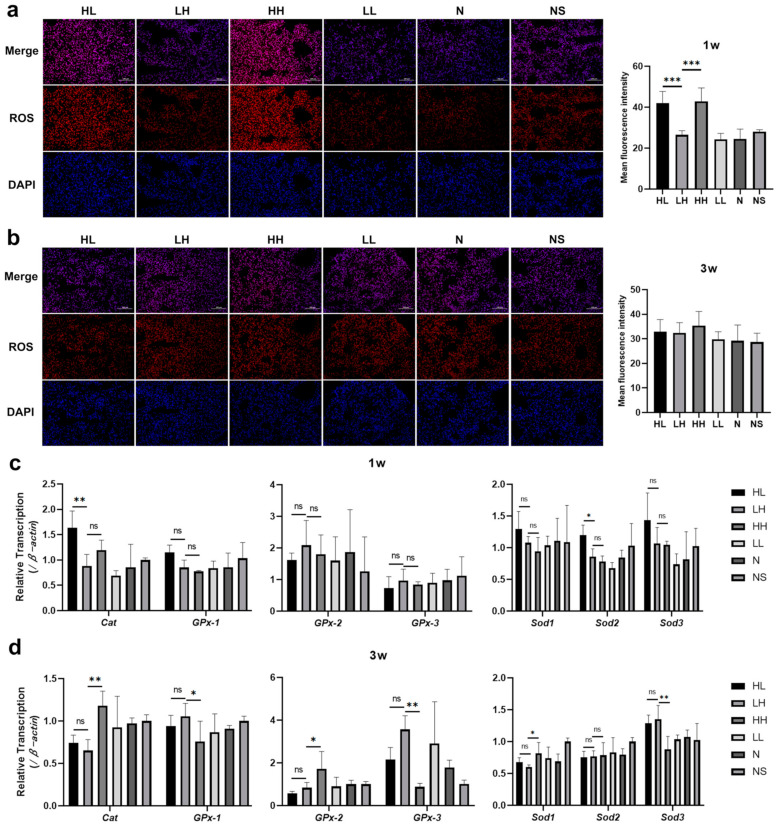
The anti-Dsg 1 antibody induced an increase in oxidative stress in the lungs of mice. (**a**) ROS fluorescence staining and mean fluorescence intensity of lung tissue after 1 week; (**b**) fluorescence staining and mean fluorescence intensity of ROS in lung tissue after 3 weeks; (**c**) changes in the expression levels of ROS metabolism-related genes in the lung tissue of mice at 1 week; (**d**) Changes in the expression levels of ROS metabolism-related genes in lung tissue of mice after 3 weeks. 1w, 1 week after the establishment of the pemphigus mouse model; 3w, 3 weeks after the establishment of the pemphigus mouse model; ROS, reactive oxygen species (red); DAPI, nucleus (blue). Data were analyzed by a one-way ANOVA with Bonferroni’s post hoc test for multiple comparisons. The histogram represents the mean ± standard deviation; ns—no significance, * *p* < 0.05, ** *p* < 0.01, and *** *p <* 0.001.

Oxidative stress was considered to play a key role in the development of ILD. We took the frozen sections of lung tissue of the mice in each group after modeling for ROS fluorescence staining and found that the level of ROS in the lung tissue of mice in the HL and HH groups increased 1 week after modeling, with significantly higher values compared to other groups (Figure 3a). However, there was no significant difference in ROS levels in the lung tissues of mice in each group at 3 weeks after modeling (Figure 3b). Catalase (*Cat*), the glutathione peroxidase family (*GPx*), and the superoxide dismutase family (*Sods*) are genes that scavenge ROS in the body. Subsequent analysis of gene expression levels related to ROS metabolism revealed that there was no statistically significant variance in the expression of ROS clearance-related genes between the HH and LH groups following 1 week of modeling. However, the expression levels of the *Cat* and *Sod2* genes in the lung tissue of the HL group were notably elevated compared to those in the LH group (Figure 3c). After 3 weeks of modeling, there was no statistically significant difference observed in the expression of ROS scavenging-related genes between the HL and LH groups. However, the *Cat*, *GPx-2*, and *Sod1* genes in the lung tissue of the HH group showed upregulation compared to the LH group, while the *GPx-1*, *GPx-3*, and *Sod3* genes exhibited downregulation (Figure 3d). The findings suggest that the pulmonary interstitial inflammation triggered by subcutaneous administration of the anti-Dsg 1 antibody in mice may be associated with the deleterious effects of anti-Dsg 1 antibody deposition in the alveolar epithelium, leading to elevated levels of ROS in lung tissue. This alteration in the expression levels of genes related to ROS metabolism may serve as a significant factor contributing to the observed increase in ROS levels.

### 2.3. H_2_ Improved Interstitial Lung Disease in P-ILD Mice

**Figure 4:**
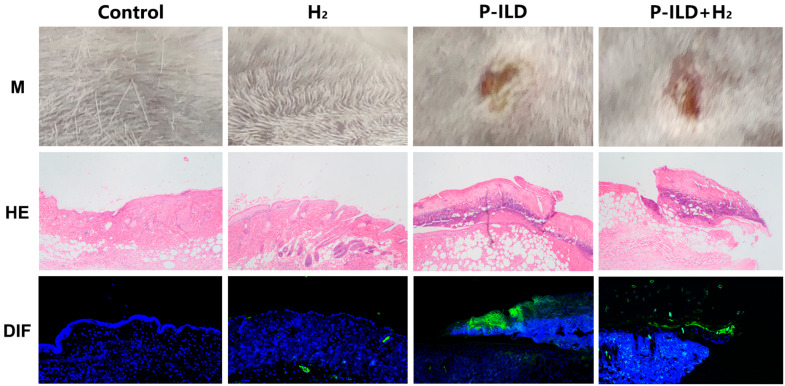
The pemphigus mouse model was successfully established in the model group and the treatment group. M shows the effect on mouse skin; HE shows hematoxylin-eosin staining of mouse skin (microscope magnification of 100×); DIF shows the direct immunofluorescence staining of mouse skin (fluorescence microscope magnification of 40×). IgG deposition (green); DAPI, nucleus (blue).

In order to investigate the therapeutic potential of hydrogen molecules, a mouse model of P-ILD was developed. Both the model group and the treatment group exhibited skin erosion and crusting. Histopathological analysis confirmed the successful establishment of the pemphigus mouse model (Figure 4).

**Figure 5:**
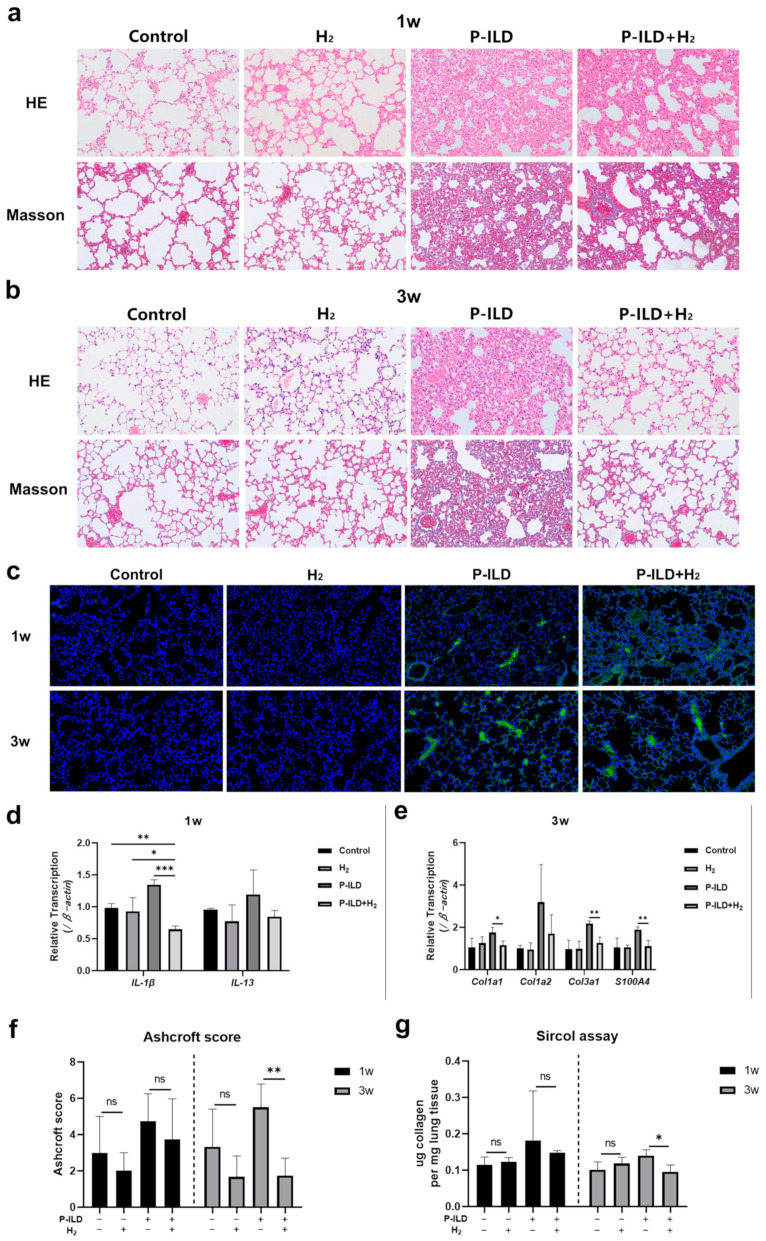
H_2_ improved interstitial lung disease in P-ILD mice. (**a**) H_2_ inhibited pulmonary inflammation in P-ILD mice; (**b**) H_2_ inhibited pulmonary fibrosis in P-ILD mice; (**c**) H_2_ did not affect IgG deposition in the lungs of P-ILD mice (fluorescence microscope magnification of 20×); (**d**) RT-qPCR was used to detect the changes of *IL-1β* and *IL-13* gene expression in the lung tissue of mice after 1 week; (**e**) RT-qPCR was used to detect the changes of *S100A4*, *Col1a1*, *Col1a2* and *Col3a1* gene expression in lung tissue of mice after 3 weeks; (**f**) Ashcroft score of lung tissue in the mice; (**g**) Collagen content in the lung tissue of mice from each group was detected by a Sircol assay. IgG deposition (green); DAPI, nucleus (blue). Data were analyzed by a one-way ANOVA with Bonferroni’s post hoc test for multiple comparisons. The histogram represents mean ± standard deviation; ns, no significance, * *p* < 0.05, ** *p* < 0.01, and *** *p* < 0.001.

Subsequently, the mice were administered either normal water or hydrogen-rich water for a duration of 1 week, following which, lung tissue samples from each group were collected for histopathological examination. The study revealed that the lung tissue of mice in the model group exhibited significant destruction, characterized by extensive infiltration of the inflammatory cells and pronounced inflammatory reactions. In contrast, the treatment group demonstrated a notably attenuated pulmonary inflammatory response compared to the model group, with moderate thickening of the alveolar septum and reduced inflammatory cell infiltration (Figure 5a). HE and Masson staining revealed that the P-ILD mice exhibited pronounced pulmonary fibrosis changes after 3 weeks, characterized by the extensive deposition of collagen fibers in the lung tissue and the formation of inter-tissue cavities, indicative of a classic ‘honeycomb lung’ phenotype (Figure 5b). The administration of hydrogen water to P-ILD mice significantly ameliorated these pathological alterations, resulting in only mild to moderate thickening of the alveolar or bronchial walls, the preservation of alveolar structure without significant disorganization, and reduced collagen deposition (Figure 5b). Immunofluorescence staining of lung tissues sampled from mice at 1 and 3 weeks revealed IgG deposition in both the model and treatment groups. Consequently, the introduction of hydrogen-rich water to the mice had not altered the deposition of IgG in their lungs (Figure 5c).

Subsequent analysis of the genes associated with lung inflammation revealed that the expression of *IL-1β* in the treatment group markedly decreased compared to both the model group and the control group, as well as the hydrogen-rich water group. Additionally, the expression of *IL-13* in the treatment group was lower than that in the model group but was similar to the levels observed in the control group and the hydrogen water group (Figure 5d). The expression of collagen genes (*Col1a1*, *Col1a2*, *Col3a1*) and profibrotic genes (*S100A4*) in the lung tissue of the model group significantly increased, while hydrogen molecules may have significantly reduced the expression of the *Col1a1*, *Col3a1*, and *S100A4* genes (Figure 5e). The Ashcroft score and Sircol assay were further used to semi-quantitatively/quantitatively evaluate the degree of lung fibrosis in each group of mice. It was found that the collagen content of the treatment group was comparable to that of the control group and the hydrogen water group at 3 weeks, and was significantly lower than that of the model group (Figure 5f,g).

The findings suggested that hydrogen molecules might effectively suppress the progression of pulmonary inflammation and fibrosis in P-ILD mice. Furthermore, there were no significant differences observed in histopathological manifestations, gene expression levels, or lung collagen content between the hydrogen water group and the control group, indicating that hydrogen did not adversely impact the physiological functioning of mouse lungs.

### 2.4. H_2_ Inhibited Oxidative Stress in the Lungs of P-ILD Mice

**Figure 6:**
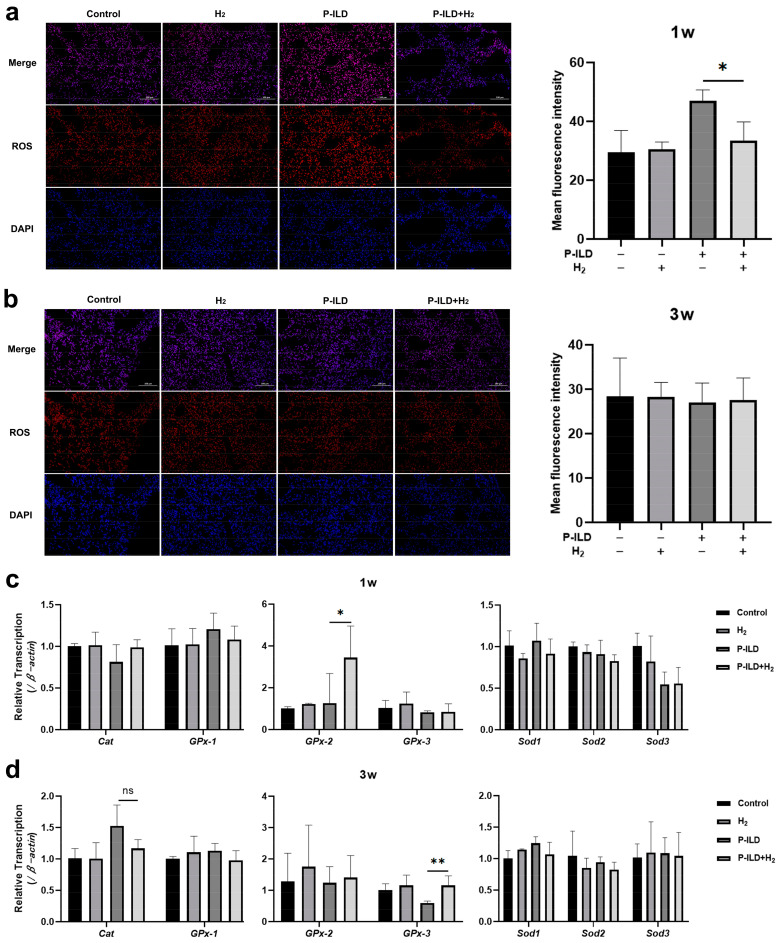
H_2_ inhibited oxidative stress in the lungs of P-ILD mice. (**a**) ROS fluorescence staining and mean fluorescence intensity of lung tissue after 1 week; (**b**) fluorescence staining and mean fluorescence intensity of ROS in the lung tissue of mice after 3 weeks; (**c**) changes in the expression levels of ROS metabolism-related genes in the lung tissue of mice after 1 week; (**d**) changes in the expression levels of ROS metabolism-related genes in the lung tissue of mice at 3 weeks. 1w, 1 week after the establishment of the pemphigus mouse model; 3w, 3 weeks after the establishment of the pemphigus mouse model; ROS, reactive oxygen species (red); DAPI, nucleus (blue). Data were analyzed by a one-way ANOVA with Bonferroni’s post hoc test for multiple comparisons. The histogram represents mean ± standard deviation; ns—no significance, * *p* < 0.05, and ** *p* < 0.01.

Following 1 week of oral hydrogen water administration, an evaluation of ROS fluorescence staining in the frozen lung tissue sections of mice revealed a significant reduction in ROS levels within the treatment group compared to the model group (Figure 6a). However, after 3 weeks of administration, no significant difference in ROS levels was observed between the two groups (Figure 6b). Notably, the ROS levels in the control group and the hydrogen water group remained comparable at both time points, suggesting that the hydrogen molecules did not disrupt the physiological redox reactions in vivo. Subsequent analysis of the gene expression related to ROS scavenging revealed that the expression of the glutathione peroxidase *GPx-2* gene in the lung tissue of mice in the treatment group was upregulated after 1 week, while the *GPx-3* gene in the lung tissue of mice in the model group was downregulated after 3 weeks (Figure 6c,d).

## 3. Discussion

Previous research has demonstrated the presence of pulmonary interstitial changes in pemphigus mice following the subcutaneous administration of serum IgG derived from pemphigus patients [5]. However, our investigation involving the subcutaneous injection of serum IgG with varying anti-Dsg 1/3 antibody titers into BALB/cJGpt mice revealed that the development of interstitial lung disease was exclusive to mice in the HL and HH groups. Mice in the LH group did not exhibit notable pulmonary interstitial inflammation or fibrosis alterations at either the 1-week or 3-week post-modeling time points. It can be inferred from this that the subcutaneous administration of the anti-Dsg 3 antibody does not effectively induce the development of a P-ILD mouse model, with the anti-Dsg 1 antibody potentially playing a significant role in the induction of interstitial inflammatory changes in the lungs of mice. Furthermore, the immunofluorescence staining of mouse lung tissues demonstrated IgG deposition in the HL and HH groups after 1 and 3 weeks, whereas no such deposition was observed in the LH group. This observation suggests that the anti-Dsg 1 antibody may interact with the antigens present in mouse lung epithelial tissues, leading to the formation of immune complexes and subsequent damage to alveolar epithelial cells.

The results of RT-qPCR analysis indicated a notable upregulation in the expression of the pro-inflammatory cytokine *IL-1β* and *IL-13* genes in the lung tissue of P-ILD mice after 1 week. Subsequently, a significant increase in the expression of the pro-fibrotic genes *α-SMA*, *S100A4*, and *TGF-β*, as well as in the collagen genes *Col1a1*, *Col1a2*, and *Col3a1* was observed after 3 weeks. The findings suggest that IL-1β and IL-13 may play a crucial role in the early stages of ILD, potentially activating myofibroblasts through an interaction with fibroblast surface receptors [24]. IL-1β induces the activation of ROS-expressing neutrophils, which can further damage the epithelial cells [25]. IL-1β also promotes the production of TGF-β1, an important profibrotic cytokine that triggers fibroblast proliferation and activation [25]. TGF-β1 can induce epithelial-to-mesenchymal transition, transforming the phenotype of lung epithelial cells into the mesenchymal cell phenotype and forming fibroblast-like epithelial cells, which is characterized by the increased expression of the mesenchymal cell marker protein α-SMA [24]. IL-13 can also directly activate fibroblasts independently of TGF-β1 [26]. Fibroblasts and fibroblast-like epithelial cells with interstitial cell characteristics secrete a large amount of extracellular matrix components such as collagen, resulting in collagen deposition in the lung and fibrosis [24].

The escalation of ROS levels can be attributed to an imbalance between pro-oxidant and antioxidant mechanisms within the body, resulting in a diminished capacity to neutralize ROS [27]. Here, we found that the level of ROS in the lungs of P-ILD mice increased, which was consistent with the results observed in the bleomycin-induced ILD mouse model and rheumatoid arthritis-related ILD mouse model, further verifying that oxidative stress is an important mechanism leading to ILD [28,29]. ROS metabolism-related enzymes, such as Cat, GPx, and Sods, are key components of the antioxidant system [10]. The variations in the expression of genes related to ROS scavenging between different groups in the various stages of inflammation and fibrosis in P-ILD suggest the potential effects of anti-Dsg 3 antibodies on oxidative stress induced by anti-Dsg 1 antibodies. Additionally, alterations in the expression levels of genes involved in ROS metabolism may contribute to elevated ROS levels.

**Figure 7:**
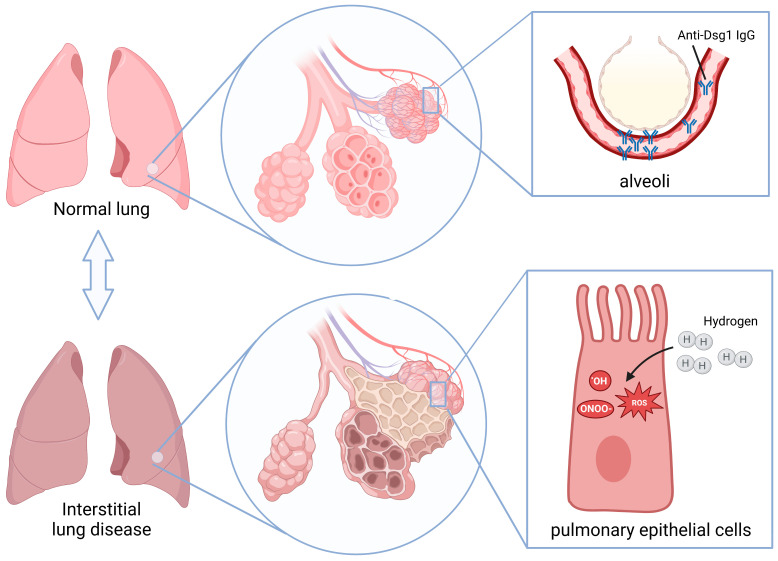
Pathogenesis of P-ILD and the therapeutic role of H_2_. Anti-Dsg 1 antibodies in the serum of pemphigus patients target the alveolar epithelium, inducing injury and triggering interstitial inflammation and, subsequently, pulmonary fibrosis. Molecular hydrogen exerts therapeutic effects by suppressing oxidative stress in the early inflammatory phase of P-ILD. Schematics were produced using BioRender (https://www.biorender.com/).

To date, numerous studies have demonstrated the therapeutic potential of hydrogen molecules in treating various lung diseases, such as acute lung injury, chronic obstructive pulmonary disease, asthma, lung cancer, and pulmonary hypertension [30,31,32,33,34]. Yasuhiro et al. found that hydrogen molecules could inhibit the inflammatory response and fibrosis by reducing the levels of lipid peroxide in serum and 8-hydroxydeoxyguanosine in the lung tissue of rheumatoid arthritis-related ILD mice, thereby reducing the expression of inflammatory factors such as TNF-α, IL-6, and TGF-β in the lungs [28]. Li Gao et al. found that hydrogen molecules reversed epithelial-mesenchymal transition by reducing the production of ROS and inhibiting the expression of TGF-β, thereby reducing extracellular matrix deposition and ultimately preventing the production of pulmonary fibrosis [35]. In this study, we investigated the therapeutic impact of hydrogen molecules on pemphigus-associated interstitial lung disease, using a mouse model (Figure 7). Consistently, our findings indicated that the sustained consumption of hydrogen water in mice with P-ILD effectively mitigated oxidative stress, leading to a reduction in pulmonary interstitial inflammation and the prevention of progression to pulmonary fibrosis.

## 4. Materials and Methods

### 4.1. Serum

Serum was collected from pemphigus patients admitted to Huashan Hospital, Fudan University (January–December 2021) and from healthy controls. Blood was drawn into serum separator tubes (BD Vacutainer^®^ SST™, Becton Dickinson, Franklin Lakes, NJ, USA), centrifuged at 1500× *g* for 10 min, and stored at −80 °C until use. Patients were stratified into four groups, based on anti-Dsg 1/3 antibody titers (≥100 U/mL: high; ≤10 U/mL: low; Table 1). Ethics approval (KY2016-398, 23 January 2017) and informed consent were obtained.

### 4.2. Animals

Male SPF BALB/cJGpt mice (6 weeks old; Jiangsu Jicui Yaokang Biotechnology Co., Ltd., Jiangsu, China; Strain Code: T001458) were housed under controlled conditions (22 ± 1 °C, 12-h light/dark cycle) with ad libitum access to standard chow (Research Diets D12450B) and water. The mice were acclimatized for 7 days prior to the experiments.

### 4.3. Hydrogen-Rich Water (HW)

HW (1.6 ppm H_2_; Shanghai Yiqingquan Health Technology Co., Ltd., Shanghai, China) was stored in aluminum-lined bags and replaced daily to maintain its concentration, which was verified by gas chromatography (GC-2014, Shimadzu, Kyoto, Japan).

### 4.4. Serum IgG Extraction

Pooled sera were processed using Protein A Spin Plates (ThermoFisher Scientific, Waltham, MA, USA; Cat# 45200) per the manufacturer’s protocol. IgG was eluted (0.1 M glycine-HCl, pH 2.7), neutralized (1 M Tris-HCl, pH 9.0), and lyophilized (Labconco FreeZone 2.5, Kansas City, MO, USA).

### 4.5. Pemphigus Mouse Model

Mice received 1 mg/10 g body weight of IgG (dissolved in 0.9% saline (Sigma-Aldrich, St. Louis, MO, USA; Cat# S8776)) via subcutaneous injection, while the controls received saline alone. Euthanasia was performed with 5% chloral hydrate (400 mg/kg, i.p.; Sigma-Aldrich, Cat# C8383). The tissue samples were fixed in 4% paraformaldehyde (24 h; Sigma-Aldrich, Cat# 158127) or stored in RNAlater (ThermoFisher Scientific, Waltham, MA, USA, Cat# AM7020). The protocols were approved by Fudan University (IDM2021060, 2 August 2021).

### 4.6. Histopathology

Hematoxylin-eosin (HE)/Masson staining: tissue sections (4 µm) were stained with Harris hematoxylin (Sigma-Aldrich, Cat# HHS32), eosin (Cat# HT110116), and Masson reagents (Sigma-Aldrich St. Louis, MO, USA, Cat# HT15). Direct immunofluorescence (DIF): tissue sections were blocked with 3% BSA (Sigma-Aldrich St. Louis, MO, USA, Cat# A7906) and then incubated with FITC-anti-human IgG (1:100; Abcam, Cambridge, UK; Cat# ab97085) and DAPI (Sigma-Aldrich St. Louis, MO, USA, Cat# D9542).

### 4.7. Ashcroft Scoring

Lung fibrosis was scored blindly by two investigators, using the Ashcroft scale [36].

### 4.8. Real-Time Quantitative Polymerase Chain Reaction (RT-qPCR)

To assess the molecular changes associated with pulmonary fibrosis and tissue remodeling in pemphigus-associated lung lesions, we selected: interleukin-1β (*IL-1β*); interleukin-13 (*IL-13*); collagen, type I, alpha 1 (*Col1a1*); collagen, type I, alpha 2 (*Col1a2*); collagen, type III, alpha 1 (*Col3a1*); alpha-smooth muscle actin (*α-SMA*); S100 calcium-binding protein A4 (*S100A4*), and transforming growth factor-beta (*TGF-β*) as the target genes, based on their established roles in fibrotic processes and tissue remodeling [24,37]. Total RNA was extracted with TRIzol (ThermoFisher Scientific, Waltham, MA, USA, Cat# 15596026) and reverse-transcribed (High-Capacity cDNA Kit, ThermoFisher Scientific, Waltham, MA, USA, Cat# 4368813). The primers (Table 2) were synthesized by Sangon Biotech (Shanghai, China). The reactions were examined using SYBR Green Master Mix (Qiagen, Hilden, Germany; Cat# 204054) in a LightCycler480 (Roche).

### 4.9. Sircol Assay

Collagen content was quantified using the Sircol Kit (Biocolor, Carrickfergus, UK; Cat# S1000) according to the manufacturer’s instructions.

### 4.10. ROS Staining

Frozen sections incubated with DHE (10 µM; ThermoFisher Scientific, Waltham, MA, USA, Cat# D11347) and DAPI were then imaged (Nikon Eclipse Ti, Tokyo, Japan). Fluorescence intensity was analyzed via ImageJ (Version 6.0.0.260, RRID:SCR_003070), available at https://imagej.net/ij/ (accessed on 4 April 2022).

### 4.11. Intervention

**Figure 8:**
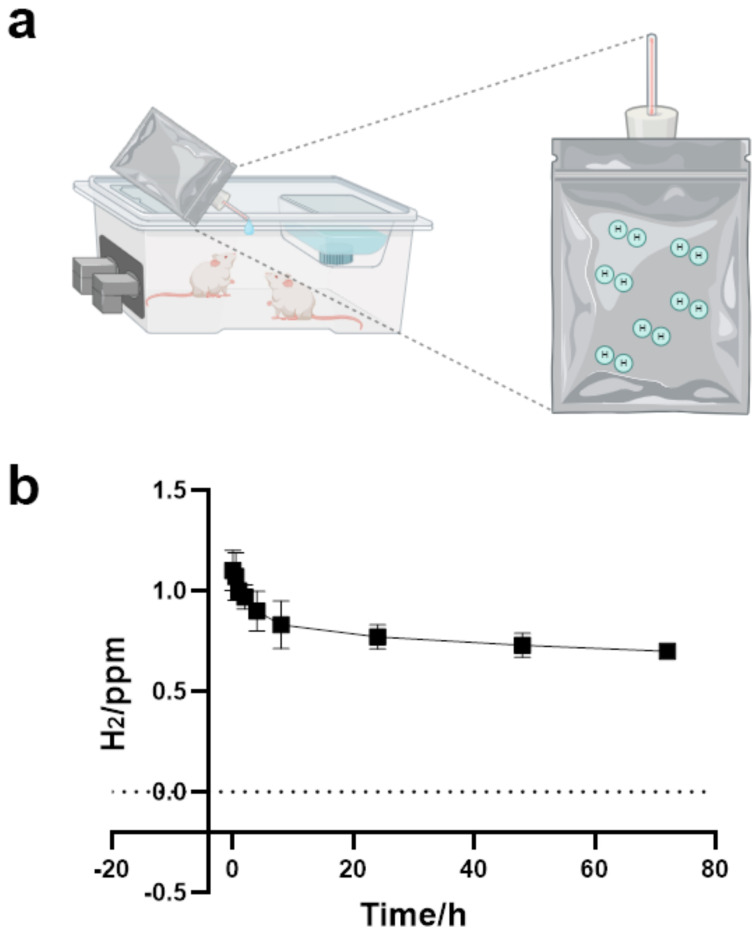
The mice were fed with hydrogen water. (**a**) Schematic diagram of the mice drinking hydrogen water; (**b**) the concentration-time curve of hydrogen molecules in aluminum foil bags. Schematics were produced using BioRender (https://www.biorender.com/).

The mice in the control group (Control) and the model group (P-ILD) were fed with normal water, and the mice in the hydrogen water group (H_2_) and the treatment group (P-ILD + H_2_) were fed with hydrogen water. During the feeding process, the aluminum foil bag containing hydrogen water was directly connected to the automatic water dispenser, and the hydrogen water was replaced once a day to ensure that the mice could drink hydrogen water containing sufficient hydrogen molecular concentrations at any time (Figure 8).

### 4.12. Statistical Analysis

Continuous variables were expressed as mean ± standard deviation. For comparisons between multiple groups, a one-way ANOVA followed by Bonferroni’s post hoc test was applied. *p* < 0.05 was considered to be statistically significant. All statistical analyses were conducted using IBM SPSS Statistics (Version 20, RRID:SCR_019096) and all figures were mapped using GraphPad Prism (Version 8.0.2, RRID:SCR_002798).

## 5. Conclusions

In summary, we found that anti-Dsg 1 antibodies in patients with pemphigus would be deposited in the lungs of mice, causing damage and inducing oxidative stress, which, in turn, led to the increased secretion of various pro-inflammatory factors and inflammatory reactions. It promoted the transformation and proliferation of lung fibroblasts, produced a large amount of collagen, led to the remodeling and deposition of the extracellular matrix, and finally formed pulmonary fibrosis. H_2_ could inhibit the early interstitial inflammatory response by reducing oxidative stress in the lungs of P-ILD mice, thereby preventing its development into pulmonary fibrosis. These results suggest that H_2_ treatment may hold the potential for managing P-ILD in pemphigus patients. Further studies are warranted to explore the clinical applicability and safety of H_2_ therapy in P-ILD and to elucidate its underlying molecular mechanisms.

## Figures and Tables

**Table 1:** Groups of serum samples.

Groups	Anti-Dsg 1 Antibody Titers (0.1–20 U/mL)	Anti-Dsg 3 Antibody Titers (0.1–20 U/mL)
Anti-Dsg 1^H^/3^L^ (HL)	≥100	≤10
Anti-Dsg 1^L^/3^H^ (LH)	≤10	≥100
Anti-Dsg 1^H^/3^H^ (HH)	≥100	≥100
Anti-Dsg 1^L^/3^L^ (LL)	≤10	≤10
Normal healthy (N)	≤10	≤10

**Table 2:** Primer sequences.

Name	Species	Sequence (5′ to 3′)
*α-SMA*-F	Mouse	GAGCGTGGCTATTCCTTCGT
*α-SMA*-R	Mouse	GCCCATCAGGCAACTCGTAA
*β-actin*-F	Mouse	GGCTGTATTCCCCTCCATCG
*β-actin*-R	Mouse	CCAGTTGGTAACAATGCCATGT
*Col1a1*-F	Mouse	GGTCCACAAGGTTTCCAAGG
*Col1a1*-R	Mouse	GCTGTTCCAGGCAATCCAC
*Col1a2*-F	Mouse	GGACCCGTTGGCAAAGATG
*Col1a2*-R	Mouse	CACCAGGAGGACCAGGAG
*Col3a1*-F	Mouse	GAGGAAACAGAGGTGAAAGAGG
*Col3a1*-R	Mouse	CAGCAATGGCAGCAGCAC
*IL-1β*-F	Mouse	AAGGAGAACCAAGCAACGACAAAA
*IL-1β*-R	Mouse	TGGGGAACTCTGCAGACTCAAACT
*IL-13*-F	Mouse	TGAGCAACATCACACAAGACC
*IL-13*-R	Mouse	GGCCTTGCGGTTACAGAGG
*S100A4*-F	Mouse	TGAGCAACTTGGACAGCAACA
*S100A4*-R	Mouse	CTTCTTCCGGGGCTCCTTATC
*TGF-β*-F	Mouse	ATTCCTGGCGTTACCTTGG
*TGF-β*-R	Mouse	CCTGTATTCCGTCTCCTTGG
*Cat-*F	Mouse	AGCGACCAGATGAAGCAGTG
*Cat-R*	Mouse	TCCGCTCTCTGTCAAAGTGTG
*GPx-1-*F	Mouse	AGTCCACCGTGTATGCCTTCT
*GPx-1-*R	Mouse	GAGACGCGACATTCTCAATGA
*GPx-2-*F	Mouse	GAGCTGCAATGTCGCTTTCC
*GPx-2-*R	Mouse	TGGGTAAGACTAAAGGTGGGC
*GPx-3-*F	Mouse	CCTTTTAAGCAGTATGCAGGCA
*GPx-3-*R	Mouse	CAAGCCAAATGGCCCAAGTT
*Sod1-*F	Mouse	AACCAGTTGTGTTGTCAGGAC
*Sod1-*R	Mouse	CCACCATGTTTCTTAGAGTGAGG
*Sod2-*F	Mouse	CAGACCTGCCTTACGACTATGG
*Sod2-*R	Mouse	CTCGGTGGCGTTGAGATTGTT
*Sod3-*F	Mouse	CCTTCTTGTTCTACGGCTTGC
*Sod3-*R	Mouse	GCGTGTCGCCTATCTTCTCAA

## Data Availability

Data in this study can be made available upon request to the corresponding author, Xiaoqun Luo (luoxiaoqun913@126.com).

## Abbreviations

The following abbreviations are used in this manuscript:

Cat	catalase
Col1a1	collagen, type I, alpha 1
Col1a2	collagen, type I, alpha 2
Col3a1	collagen, type III, alpha 1
DIF	direct immunofluorescence
Dsg	desmoglein
GPx	glutathione peroxidase
H_2_	hydrogen molecules
HH	anti-Dsg1^H^/3^H^
HL	anti-Dsg1^H^/3^L^
HW	hydrogen-rich water
IL-13	interleukin-13
IL-1β	interleukin-1β
IL-6	interleukin-6
ILD	interstitial lung disease
LH	anti-Dsg1^L^/3^H^
LL	anti-Dsg1^L^/3^L^
N	normal healthy people
NAC	N-Acetylcysteine
NS	normal saline
P-ILD	pemphigus associated interstitial lung disease
ROS	reactive oxygen species
RT-qPCR	real-time quantitative polymerase chain reaction
S100A4	S100 calcium binding protein A4
Sod	superoxide
TGF-β	transforming growth factor-beta
TNF-α	tumor necrosis factor-alpha
α-SMA	α-smooth muscle actin
